# Fourteen complete genomes of nematode-mutualistic *Xenorhabdus* bacteria reveal their high polylysogeny

**DOI:** 10.1128/mra.00764-26

**Published:** 2026-10-16

**Authors:** Ryan M. Awori, Farrah Bashey, Heidi Goodrich-Blair

**Affiliations:** 1 Department of Microbiology, University of Tennessee 4292, Knoxville, Tennessee, USA; 2 Elakistos Biosciences, Nairobi, Kenya; 3 Department of Biology, Indiana University, Bloomington, Indiana, USA; Portland State University, Portland, Oregon, USA

**Keywords:** complete genome assembly, prophages, polylysogeny, *Xenorhabdus* bacteria, animal host-microbe interactions

## Abstract

Here, we report complete genome assemblies of 14 *Xenorhabdus* bacterial strains, previously isolated from their symbiotic *Steinernema* nematode hosts. These complete genomes enabled identification of complete prophage/phage-like loci that average eight per genome, indicative of high polylysogeny among *Xenorhabdus* bacteria.

## ANNOUNCEMENT

**Fig 1:**
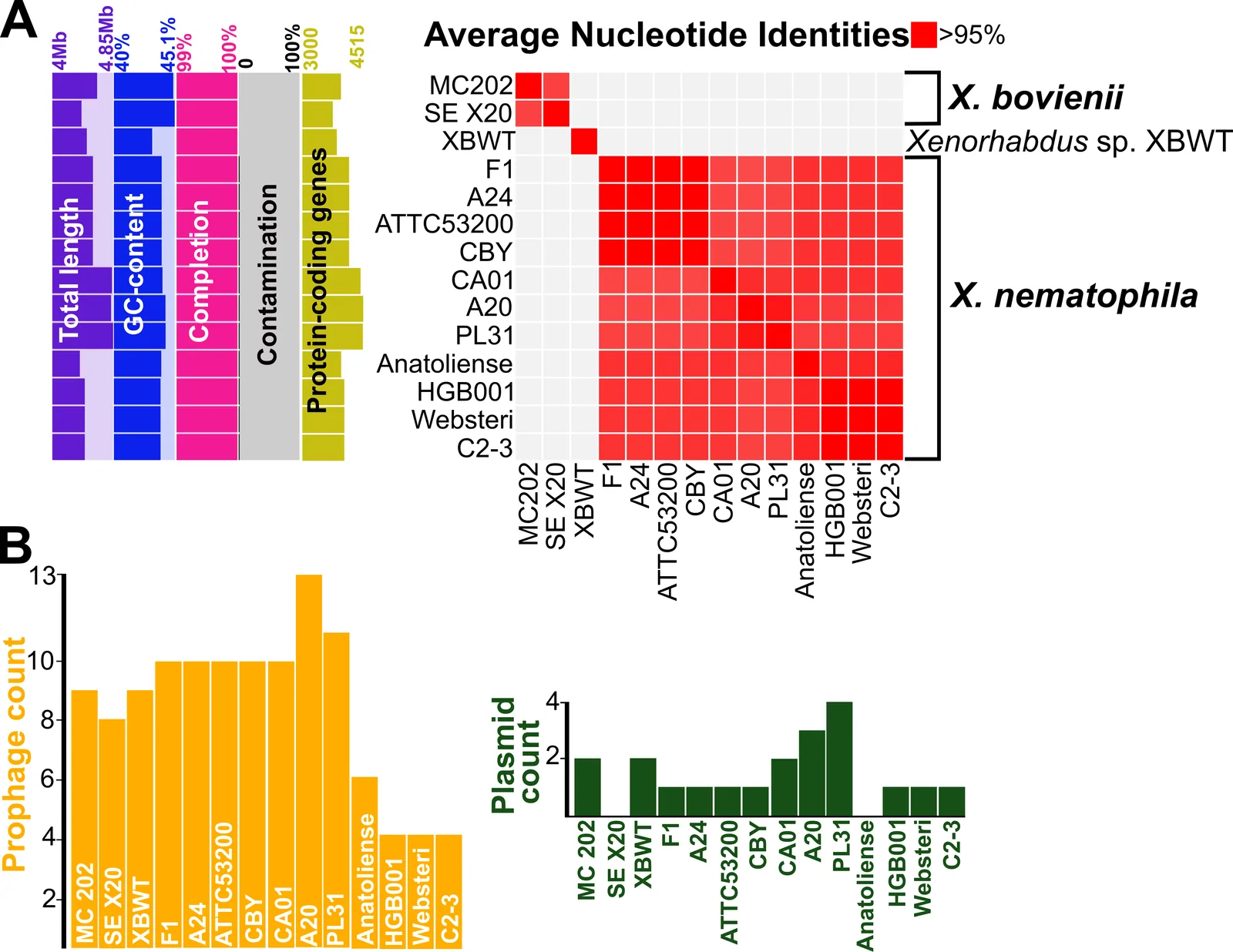
Characteristics of 14 *Xenorhabdus* genomes assembled in this study. (**A**) All genomes had 100% completion and less than 1.8% contamination. GC content varied between species, but within a species, the variation did not exceed 1%. Genome length ranged from 4.38 to 4.85 Mb. (**B**) Overall relatedness of genomes based on pairwise average nucleotide identities (P-ANI). Red indicates P-ANI values above the 95% threshold for conspecific strains (5). For the 11 X. *nematophila* species, P-ANI ranged from 99.99% to 98.51%. For the two *X. bovienii* species, their P-ANI was 98.6%.

*Steinernema* nematode-*Xenorhabdus* bacteria mutualists are entomopathogenic (1). *Xenorhabdus* genomes have high prophage/phage-like gene content, which largely accounts for their varying protein-coding gene number (Fig. 1) (2). These loci average 35 kilobases and often span contigs in fragmented assemblies (2), impeding analyses of their roles in genome evolution and function (3). We sequenced and assembled 14 complete *Xenorhabdus* genomes (Fig. 1, Table 1), enabling confident identification of 118 complete prophages/phage-like loci and supporting the conclusion that with ~8.4 prophages per genome, the *Xenorhabdus* genus has nearly unmatched polylysogeny (2, 4).

**TABLE 1:** Genome assembly summary data^*a*,*b*^

Strain	Genome size	Chromosome size	Reads	Read *N*_50_	Mean read length	Bases (G)	Depth (*x*)	Completion (%)	Contamination (%)	GC (%)	Nematode source	Ref/source^*a*^
*Xenorhabdus nematophila*												
ATTC53200	4,568,257	4,541,791	248,672	4,917	3,299	0.82	179	100	1.4	44	*S. carpocapsae*	(6, 7)
A20	4,823,449	4,789,815	428,230	7,216	4,212	1.8	373	100	1.4	44.3	*S. carpocapsae* (Portugal)	(8)
A24	4,568,103	4,541,637	955,202	9,048	5,290	5	1,096	100	1.4	44	*S. carpocapsae* Agritos (Russia)	(8, 9)
Anatoliense*	4,385,815	4,360,943	813,249	2,933	2,122	1.73	394	100	1.4	43.9	*S. anatoliense* (Jordan)	(10)*
CBY	4,568,256	4,541,790	708,651	7,864	4,616	3.3	723	100	1.4	44	*S. carpocapsae* (China)	(8)
CA01	4,835,657	4,663,316	426,703	8,041	4,897	2.09	432	100	1.4	44.1	*S. carpocapsae* (Canada)	(8)
C2-3*	4,454,936	4,295,114	536,999	9,157	5,429	2.9	648	100	1.4	43.9	*S. carpocapsae* (South Korea)	(11)*
F1*	4,569,593	4,543,127	381,381	10,656	6,116	2.3	504	100	1.4	44	*S. carpocapsae* (France)	(9)*
HGB001	4,454,940	4,295,120	465,675	10,804	5,108	2.38	534	100	1.4	43.9	*S. carpocapsae* ALL (Georgia, USA)	(12)
PL31	4,852,720	4,769,017	842,062	10,420	6,107	5.14	1,142	100	1.4	44.3	*S*. *carpocapsae* (Poland)	(8)
Websteri*	4,454,937	4,295,115	187,881	3,011	2,150	0.4	89	100	1.4	43.9	*S. websteri* (Jordan)	(10)*
*Xenorhabdus bovienii*												
MC202*	4,627,923	4,526,038	261,657	10,241	5,037	1.3	284	100	0	44.9	*S. affine* (Indiana, USA)	(13)*
SE_X20	4,408,700	4,408,700	335,316	13,133	6,681	2.24	502	100	0	45.1	Unknown	C. Ensign
*Xenorhabdus* sp.												
XBWT	4,483,539	4,321,989	278,959	8,474	5,558	1.55	345	100	0	43.2	*S. weiseri* (Türkiye)	P. Stock

^*a*^ Asterisks indicate that previously published, incomplete/uncircularized genome assemblies are available. Read N_50_, the shortest read among the minimum number of reads that contain 50% of the base pairs sequenced; Gbp, Gigabase pairs; Depth, total number of basepairs sequenced/genome size; GC, guanine-cytosine content; Ref, Published article on the provenance of the strain.

^*b*^ Descriptive metrics for the sequencing data of each of 14 strains are provided, including genome size, chromosome size, reads, *N*_50_, mean read length, bases, depth, completion, contamination, and GC content. The nematode host and reference/source for each strain are also provided.

After streaking from cryostocks onto agar and incubation for 2–3 days at 30°C, single colonies were inoculated and grown overnight in lysogeny broth (LB) medium. DNA was extracted with Purelink Genomic DNA extraction kits (Invitrogen). Samples had >1.7 *A*_260_/*A*_280_ values and DNA concentrations of 100–734 ng/μL (Nanodrop One Spectrophotometer, Thermo Scientific). Samples were outsourced for sequencing (Plasmidsaurus, USA) using Oxford Nanopore tagmentation amplification-free v14 chemistry (SDK-RAD114) libraries and R10 flow cells, with these Dorado base calling parameters: dna_r10.4.1_e8.2_400bps_sup@v4.3.0, with default Q10 quality filtering.

For each read set, subsampling was performed using Autocycler v.0.6.2 (14) with these parameters: count: 2/4; min_read_depth: 25/50/100; genome_size: 4.5m. For each read subsample, Flye v2.9.6 (15)/Canu v2.3 (16)/Hifiasm v.0.25 (17) on Galaxy platforms (https://www.usegalaxy.org, usegalaxy.eu, and usegalaxy.org.au) (18) was used for genome assembly using default settings for high accuracy Oxford Nanopore reads. At least four assemblies per sample were obtained and used for Autocycler compress then cluster programs with parameter max-contigs: 50/100/150/200. Outputs were fed into Autocycler trim, then Autocycler resolve, and finally Autocycler combine was used to create the final fasta file which was used as input in Dnappler v.1.3 (19) for rotation of replicons under default settings. Prophages were identified with Genomad v.1.9 (20) on Galaxy using default parameters, which was also used to additionally identify plasmid replicons. Completion and contamination levels were determined in Anvio-dev (21) as previously described (2). Average nucleotide identities were calculated using fastANI. Assemblies, including plasmids, were annotated with PGAP (22). The outputs from genome analyses, plasmid and prophage identification are available at https://doi.org/10.5281/zenodo.22836655.

## Data Availability

Accession numbers are GCA_059072775.1, GCA_059639835.1, GCA_059639815.1, GCA_059639915.1, GCA_059639895.1, GCA_059639875.1, GCA_059639875.1, GCA_059072445.1, GCA_059072435.1, GCA_059072295.1, GCA_059072925.1, GCA_059072915.1, GCA_059072745.1, GCA_059076135.1, and GCA_059072735.1. Sequence reads accession numbers are SRX33985650, SRX33977297, SRX33977140, SRX33977055, SRX33976834, SRX33976833, SRX33975850, SRX33975569, SRX33973165, SRX33972839, SRX33971767, SRX33968637, SRX33968555, and SRX33968236.
